# Re-evaluation of the Carcinogenic Significance of Hepatitis B Virus Integration in Hepatocarcinogenesis

**DOI:** 10.1371/journal.pone.0040363

**Published:** 2012-09-04

**Authors:** Suzhen Jiang, Ziwei Yang, Weijie Li, Xiaojun Li, Yongfeng Wang, Jiangbo Zhang, Chunhui Xu, Pei-Jer Chen, Jinlin Hou, Malcolm A. McCrae, Xiangmei Chen, Hui Zhuang, Fengmin Lu

**Affiliations:** 1 Department of Microbiology, School of Basic Medical Sciences, Peking University Health Science Center, Beijing, Beijing, China; 2 Department of Infectious Disease, Nanfang Hospital, Southern Medical University, Guangzhou, Guangdong, China; 3 Department of Infectious Disease, Linyi People's Hospital, Linyi, Shandong, China; 4 Hepatitis Research Center, National Taiwan University and Hospital, Taipei, Taiwan; 5 Department of Biological Sciences, University of Warwick, Coventry, United Kingdom; 6 Infectious Disease Center, Peking University, Beijing, China; University of Hong Kong, Hong Kong

## Abstract

To examine the role of hepatitis B virus (HBV) integration in hepatocarcinogenesis, a systematic comparative study of both tumor and their corresponding non-tumor derived tissue has been conducted in a cohort of 60 HBV associated hepatocellular carcinoma (HCC) patients. By using Alu-polymerase chain reaction (PCR) and ligation-mediated PCR, 233 viral-host junctions mapped across all human chromosomes at random, no difference between tumor and non-tumor tissue was observed, with the exception of fragile sites (*P* = 0.0070). HBV insertions in close proximity to cancer related genes such as hTERT were found in this study, however overall they were rare events. No direct correlation between chromosome aberrations and the number of HBV integration events was found using a sensitive array-based comparative genomic hybridization (aCGH) assay. However, a positive correlation was observed between the status of several tumor suppressor genes (TP53, RB1, CDNK2A and TP73) and the number of chromosome aberrations (r = 0.6625, *P* = 0.0003). Examination of the viral genome revealed that 43% of inserts were in the preC/C region and 57% were in the HBV X gene. Strikingly, approximately 24% of the integrations examined had a breakpoint in a short 15 nt viral genome region (1820–1834 nt). As a consequence, all of the confirmed X gene insertions were C-terminal truncated, losing their growth-suppressive domain. However, the same pattern of X gene C-terminal truncation was found in both tumor and non-tumor derived samples. Furthermore, the integrated viral sequences in both groups had a similar low frequency of C1653T, T1753V and A1762T/G1764A mutations. The frequency and patterns of HBV insertions were similar between tumor and their adjacent non-tumor samples indicating that the majority of HBV DNA integration events are not associated with hepatocarcinogenesis.

## Introduction

Primary hepatocellular carcinoma (HCC) is the most common cause of cancer death in China [1]. There were an estimated 630,000 newly diagnosed HCC cases annually worldwide and more than 55% are attributed to Chinese [2]. It is estimated that more than 80% of HCC is etiologically associated with hepatitis B virus (HBV) in China [3].

As an oncogenic virus, HBV leads to HCC both directly and indirectly [4], [5]. Chronic HBV infection results in persistent inflammatory damage to hepatocytes and compensatory regeneration. During the endless cycles of hepatocyte damage and regeneration, mutations accumulate and ultimately liver malignancy occurs. Oncogenic viral proteins such as HBx and mutant large surface protein were also considered as playing direct pathogenic roles [6], [7], [8]. In addition, it has been proposed more than three decades ago that HBV DNA integration into the hepatocytes cellular genome played a causative role in hepatocarcinogenesis [9]. However, this speculation was mostly based on small scale observational studies using only tumor tissue and lacked the comparative control of adjacent non-tumor tissue [8], [10], [11], [12], [13], [14]. Therefore, analysis of adjacent non-tumor tissue and consequently a systematic investigation of this hypothesis is still required.

In this study, using both Alu-polymerase chain reaction (Alu-PCR) and cassette ligation mediated PCR (LM-PCR), we thoroughly analyzed the HBV integration events in up to 60 paired HBV-HCC tumor and adjacent non-tumor tissues, focusing on the discrepancy of integration frequency, host chromosome integration sites and the precise viral-host sequences, the mutation of the viral genes, etc. Furthermore, array-based comparative genomic hybridization (aCGH) was conducted to assay the co-relationship between HBV DNA integration and host chromosomal aberration. In addition, in order to provide a full holographic view of the pathogeneity of HBV integration in HCC, TP53 status was also considered. Through this systematic investigation, the carcinogenesis significance of HBV integration in hepatocarcinogenesis was re-evaluated.

## Materials and Methods

### Patients

Sixty HCC patients undergoing surgical operations were recruited from He'nan Cancer Hospital from 2008 to 2009 (ranging age from 30 years to 70 years, mean age = 50.7±8.46 years; male: female = 42: 18; 3 of them were HBeAg-positive and 57 were HBeAg-negative); 58 of them had accompanying liver cirrhosis (Table S1). All HCC diagnoses were confirmed pathologically and their tumor stage was determined according to the 2002 International Union Against Cancer TNM Classification System.

The patients enrolled in this study fulfilled the following criteria: hepatitis B surface antigen positive; hepatitis B virus genotype C; negative for hepatitis C virus antibody (EIA, Abbott Laboratories). Autoimmune liver disease, drug-related hepatitis, alcoholic hepatitis, and obstructive jaundice were all excluded; none of the patients had undergone interferon therapy or other anti-virus therapy. Our investigation has been conducted according to the principles expressed in the Declaration of Helsinki. This study was approved by the Ethics Committee of Peking University Health Science Center, and written informed consent was approved from all participants involved in our study. The Ethics Committees of Peking University Health Science Center approved the consent procedure.

### Sample preparation

DNAs were extracted from 60 paired frozen HCC tissues and corresponding adjacent non-tumor liver tissues using proteinase K followed by a standard phenol/chloroform extraction and ethanol precipitation method. For the aCGH study, the genomic DNAs were extracted using the Genomic DNA purification Kit (Qiagen, USA).

### Identification of viral-host junctions

LM-PCR was employed using cassette primers and primers specific to HBV sequences to amplify viral-host junctions, as previously described [11]. Alu-PCR was employed using specific primers to human Alu sequences and to HBV sequences to efficiently amplify viral-host junctions, as previously reported with modified primers HBV1 and HBV2 (Table S2) [10], [11]. The HBV specific primers were from the HBV X gene for the forward primers and the HBV preC/C gene for the reverse primers. Both LM-PCR and Alu-PCR have their own limitations. The Alu-PCR approach can only detect viral integration close to a human Alu repeat sequence. By contrast the LM-PCR method requires that there is a recognizable restriction endonuclease site not distant from the point of viral insertion. Therefore, the two methods were used in complementary for the analysis of viral integration events in each specimen.

The PCR products were subjected to agarose gel electrophoresis, and the DNA bands were cut out of the gels for subsequent sequencing. The majority of the viral-host junction sequences we got were from direct sequencing of PCR products, either from Alu-PCR or from LM-PCR. The PCR products were sub-cloned into the TA cloning vector (Genstar, Beijing, China) when direct sequencing failed. The viral-host sequences were analyzed by using the NCBI Blast tool and UCSC database hg19 to identify viral genome sequences, and to map the integration sites in the human genome.

### Detection of HBV X gene mutation in tumor and non-tumor

The sequences for the ‘free’ HBx region were detected by PCR using primers (HBV3 and HBV4, Table S2) spanning from the X gene to the preC/C gene. The approximately 1100 bp PCR products were directly sequenced. The integrated HBV X region sequences were acquired from the confirmed viral-host junction sequences.

### Analysis of HCCs tumor cell genomic instability

A total of 25 paired DNA samples derived from tumor tissues and the corresponding non-tumor tissues were prepared. Chromosome aberration was comprehensively analyzed via aCGH. In the assay, each corresponding paired adjacent non-tumor tissue DNA was used as reference DNA. The aCGH was performed by the Shanghai Bio Corporation (Shanghai, China) using the Aiglent Human Genome 244K CGH microarray that contains 236,000+ coding and non-coding human sequences, according to the manufacturer's instructions. The raw aCGH profiles extracted from Agilent Feature Extraction 10.5.1.1 were processed to identify statistically significant transitions in copy number using the aberration detection method algorithm found in Agilent DNA Analytics 4.0.

### Comprehensive analysis of the status of TP53 gene in tumor tissues

The exons 2 to 11 of TP53 were amplified using 4 independent PCR reactions. The PCR products were directly sequenced to identify mutations. Meanwhile, 9 SNP sites (rs1642785, rs17878362, rs17883323, rs1042522, rs77624624, rs2909430, rs12947788, rs12951053 and rs6503048) in this region were also analyzed for any potential loss of heterozygosity (LOH), in comparison with the corresponding non-tumor tissues. Fifty nanograms of DNA were subjected to PCR reactions using the HotStar High Fidelity Taq polymerase (Genstar, Beijing, China). The primers and PCR conditions are shown in Table S3. All amplified samples were examined by agarose gel electrophoresis to confirm successful amplification. The purified fragments were directly sequenced using the Genetic Sequencer ABI 3100 from Applied Biosystem.

Through comparing the electrophograms of the heterozygote SNP sites, LOH was defined according to the following formula: LOH index: L = (T2/T1)/(N2∶N1) (T is tumor tissue, N is the adjacent non-tumor tissue;1 and 2 are the intensities of smaller and larger alleles.). If the LOH index was less than 0.5 or more than 2.0, this defined the case as a potential LOH site. TP53 LOH was defined when there were two or more than two potential LOH sites in each tissue. This method was validated using the 25 aCGH analyzed tumor and non-tumor tissues.

### Statistical Analysis

All statistical analyses were performed using the SPSS 14.0 for windows. The χ^2^ test with Yates correction factor or Fisher's exact test was used to compare categorical variables between two groups. To study potential correlation between the number of HBV integration and chromosomal aberration number, Spearman's correlation test was used. To study the correlation between the number of tumor suppressor gene aberrations and the number of chromosomal aberration, Spearman's correlation test was used. All estimates were accompanied by a 95% confidence interval (CI), where appropriate and a P-value<0.05 was considered as being statistically significant.

## Results

### HBV DNA integration shows no difference either in frequency or chromosome distribution between tumor derived and the corresponding non-tumor derived samples

Using Alu-PCR and LM-PCR, all 5 known viral-host sequences in the PLC/PRF/5 cell line were detected [4], indicating that the methods we employed here were reliable. A total of 287 different inserted sequences were identified amongst the 88% (53/60) integration positive patients (File S1). Amongst these 101 viral-host junctions were identified in 68% (41/60) of the tumor derived samples and 186 in 72% of the (43/60) non-tumor derived samples. Multiple integration events were found in 50% (22/41) of the tumor derived and 91% (39/43) of the non-tumor derived samples. Amongst the viral-host junctions identified, 233 could be precisely mapped to chromosomes, of which 81 were from tumor derived tissues and 152 from non-tumor samples. The remaining 54 virus-host junctions could not be uniquely mapped due to repetitive or unidentified sequences. A greater number of integration events were detected in non-tumor derived specimens (2.43 per tumor tissue and 4.33 per non-tumor tissues respectively).

As expected, larger chromosomes harbored more integration events. However, when normalized to the number of integrations per 10^8^ base pairs, no obvious chromosome preference was observed in either tumor derived or in non-tumor derived samples. Amongst the 233 precisely mapped viral insertion sites, 64 were found to lie within a known fragile site [15]. The following fragile sites were found being hit more than once: FRA19B in 5 cases, FRA1A, FRA5A, and FRA7J each in 4 cases, FRA7B and FRA1L in 3 cases, FRA1F, FRA12A, FRA3D, FRA6D and FRA19A in 2 cases. Interestingly, a significant disparity was observed in the frequency with which fragile sites were mapped occurred between the tumor derived and non-tumor samples was observed (31/81 sites in tumor vs. 33/152 sites in non-tumor, *P* = 0.0070).

### Cellular gene containing areas in the human genome are the favored target site for HBV integration

**Figure 1:**
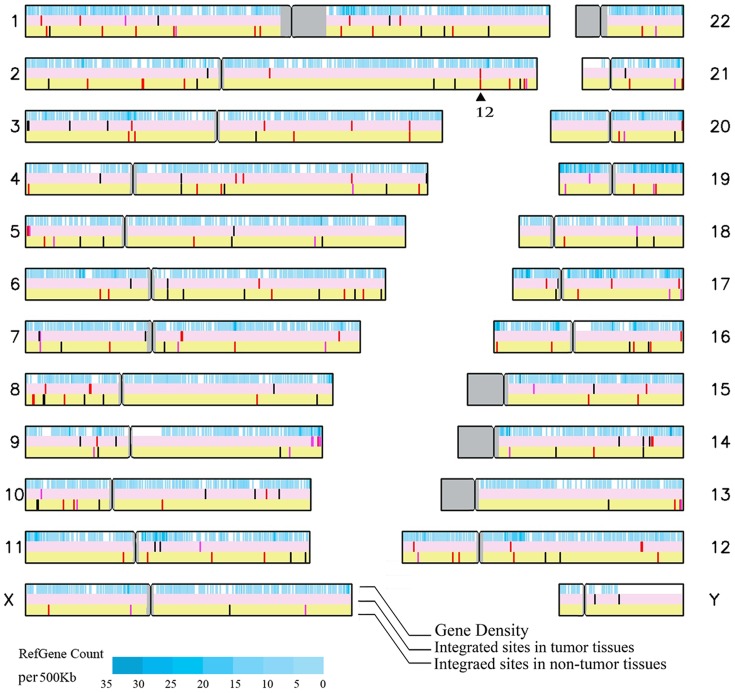
Map of the target sites of HBV integration in human chromosomes. The solid black triangle indicates 12 insertion sites within the FN1 gene. The red bars indicate target sites located within cellular genes; the pink bars indicate target sites within 10 kb upstream or downstream of cellular genes; the black bars indicate integration sites falling more than 10 kb upstream or downstream of cellular genes.

Alignment analysis using the UCSC blast revealed that 47% (110/233) of the viral insertion sites mapped were in introns (35/81 in tumor vs. 75/152 in non-tumor) and 4% (10/233) fell in exons (4/81 in tumor vs. 6/152 in non-tumor) (File S1). The remaining 48% (113/233) were mapped to non-coding regions of the human genome (43/81 in tumor vs. 70/152 in non-tumor) (File S1). In addition, 11 of 81 integration sites mapped from tumor derived samples fell within ±10 kb of transcription start sites whilst the same was true for only 15 of the 152 integration sites mapped for non-tumor derived samples. These data indicate that, promoter, exon and intron areas in the genome are the favored target sites for HBV integration. It is of course possible that direct insertion into a gene area could affect the function of the targeted gene. However, no significant differences in integration preference were observed between the tumor derived and the non-tumor derived samples (Figure 1).

Several previous reports have suggested that a number of cellular genes such as human telomerase reverse transcriptase (hTERT) and Fibronectin 1 (FN1) were favored targets of HBV integration in HCC tumor tissue [10], [13], [16]. In the present study, insertion in or around the hTERT gene was found in 3 tumor derived samples. This observation provides additional evidence that hTERT is frequently hit by HBV integration. HBV insertion directly into the FN1 gene was also found in 12 cases. However, only 2 of these were found in the tumor derived samples (Table 1). This latter observation challenges the previously proposed direct oncogenic role of such insertions in hepatocarcinogenesis. It is worthy of note that in the earlier studies on which this proposal was based they did not involve simultaneous analysis of both tumor derived and non-tumor tissue of the type undertaken in the present study.

**Table 1:** Precise locations of HBV integration events mapped to the FN1 gene.

Case No.	Location in Chr2	Orientation	Precise location	HBV break point
09HP-2	216271615	opposite	Intron	1783
09HP-9	216255166	opposite	Intron	1405
09HP-10	216289986	opposite	Exon	1821
09HP-32	216269562	opposite	Intron	1818
09HP-64	216250946	opposite	Intron	1747
09HP-65	216264921	same	Intron	1819
09HP-68	216297676	same	Intron	1825
09HP-68	216293166	same	Intron	1838
09HP-70	216293284	opposite	Promoter region	1838
325C	216251299	opposite	Exon	1940
509C	216245759	opposite	Intron	1796
414CP	216291241	opposite	Intron	1842

The chromosome locations were mapped using the UCSC database. The orientation of the cellular gene was compared with that of the integrated HBV genome: same = same direction while opposite = opposite direction.

The myeloid/lymphoid or mixed-lineage leukemia 4 (MLL4) gene has also been reported as being frequently hit by HBV integrations, however, none was found in this study [16].

### No difference in the viral break point pattern was found between tumor derived and non-tumor derived samples

Sequence analysis of the 287 inserted viral fragments revealed that 164 of them harbored partial X gene sequences and 120 harbored preC/C gene sequences, leaving 3 that contained neither the X gene nor preC/C gene sequences. As previously reported, most of the break points occurred around the DR1 site [4]. This is the first study in which large numbers of inserted preC/C gene sequences have been found in HCC patients.

**Figure 2:**
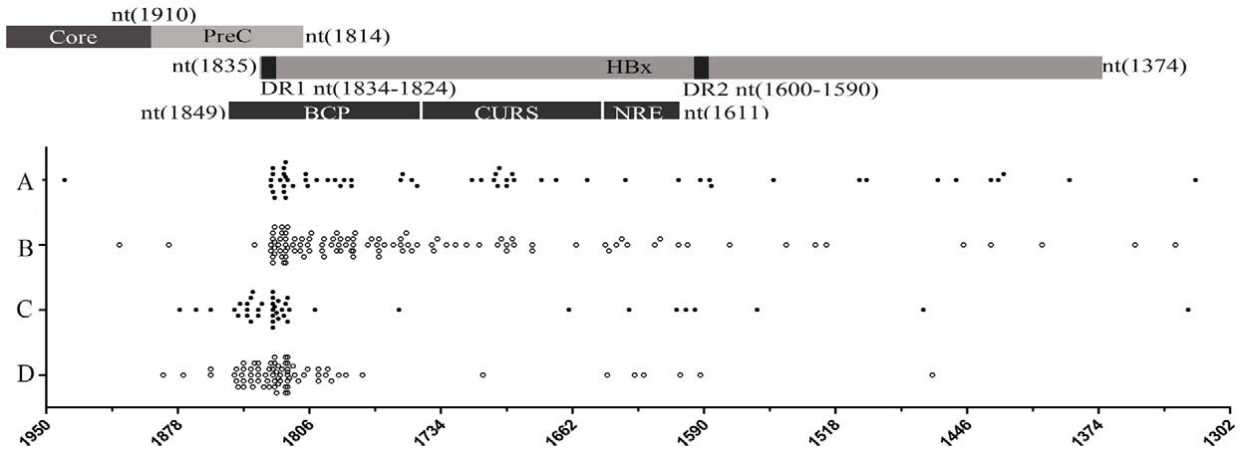
The distribution of break-points in the HBV genome in integrated viral sequences detected using different viral primers. A and B, HBV genome break-points were obtained using HBV specific primers (S1 and pUTP) lying downstream of the HBx region. C and D, HBV genome break-points were obtained using a primer (HBV1920R) lying downstream of the HBV core region. Solid and Hollow dots represent virus-cell junction sites from tumor derived and non-tumor derived samples respectively. Five of the break-points identified fell outside of the region of the viral genome shown in detail in this figure (at nt 415; nt 2784; nt 1292; nt 546; and nt 3075).

Approximately 75% of the break points mapped between nt 1601 and nt 1834 of the viral genome, with 24% (68/287) of them being located in the 5′-CTTTTT-3′ topoisomerase I motif (1820 to 1825 nt) and the DR1 region (1824–1834 nt) (Figure 2). The DR2 (1590–1600 nt) region was rarely found as a break point (Figure 2). Therefore overall the data indicated that the topoisomerase I motif and the DR1 region of the viral genome were the preferred HBV genome break-points in the mapped integration sites, but failed to reveal any difference between those from tumor derived and non-tumor derived samples.

### Comparative analysis of mutations in the inserted viral DNA failed to reveal any difference between tumor derived and non-tumor derived samples

Meta-analysis of previously published data, both our own and that of others, has shown that the number of mutations of the HBV genome (C1653T, T1753V and A1762T/G1764A) gradually increased with disease progression and correlated with hepatocarcinogenesis (Table 2) [17].

**Table 2:** Frequencies of C1653T, T1753V, or A1762T/G1764A mutations in samples from the recruited patient cohort and previously published data from patients at different stages of disease progression.

	Integration viral DNA			‘free’ HBV DNA						
	Total	Tumor	Non-tumor	Total	Tumor	Non-tumor	ASC*	CHB*	LC*	HCC*
T1653	9% (7/75)	6% (2/34)	12% (5/41)	22% (15/68)	23% (7/30)	24% (8/34)	13% (29/227)	15% (71/469)	32% (50/157)	37% (257/695)
V1753	20% (12/59)	13% (3/24)	26% (9/35)	33% (20/60)	26% (7/27)	39% (13/33)	13% (29/227)	20% (96/469)	32% (50/157)	39% (317/812)
T1762/A1764	54% (31/57)	48% (11/23)	59% (20/34)	78% (47/60)	74% (20/27)	81% (27/33)	28% (197/697)	50% (571/1145)	71% (242/343)	71% (1566/2217)
A1764					78% (21/27)	88% (29/33)				

* **from the pooled data** [**15**] **.**

Direct sequencing of PCR amplicons generated from both tumor derived and non-tumor derived integrated viral sequences (integrated group) revealed that they were carrying fewer point mutations than were found in ‘free’ non-integrated HBV genomes. The frequencies of C1653T, T1753V and A1762T/G1764A mutations found were at the same level as that seen in the serum derived samples of the CHB group (Table 2), and were significantly lower than that in the LC and HCC group (Table 2). A 1.1 kb amplicon (from nt 1264–2362) from ‘free’ non-integrated HBV DNA was successfully amplified and sequenced from a total of 30 tumor derived and 34 non-tumor derived samples (Table 2). As expected, the frequencies of C1653T, T1753V, A1762T/G1764A mutations in these ‘free’ HBV DNAs from both tumor derived and non-tumor derived samples were at the same level as that for the serum derived samples of the LC group and HCC group (Table 2). It is worth noting that the frequency of either C1653T or A1762T/G1764A mutations seen was significantly higher than that found for the integrated group (C1653T, 9% vs. 22%, *P* = 0.0110; T1762/A1764, 78% vs. 54%, *P* = 0.0060). This significant difference remained when the frequencies of the T1762 or A1764 point mutation (*P*<0.05) were separately compared.

None of 164 confirmed X region insertions sequenced carried a whole X gene, with all of the inserts having a 3′ terminal truncation. The shortest deletion found had only lost the last 8 nucleotides of the X gene. Consequently none of these integrated viral genomes retained an intact growth-suppressive effect domain which maps to nucleotides 1794–1838 of the HBV genome. Deletions of the p53-dependent transcriptional repression binding site (nt 1635–1665) and the Sp1 binding site (nt 1731–1752) were also found in 13% (22/164) and 28% (46/164) of insertions, respectively. No significant difference was found for these or the other mutations described above between integrated viral genomes in tumor derived and non-tumor derived samples.

### DNA rearrangement surrounding integration sites

**Figure 3:**
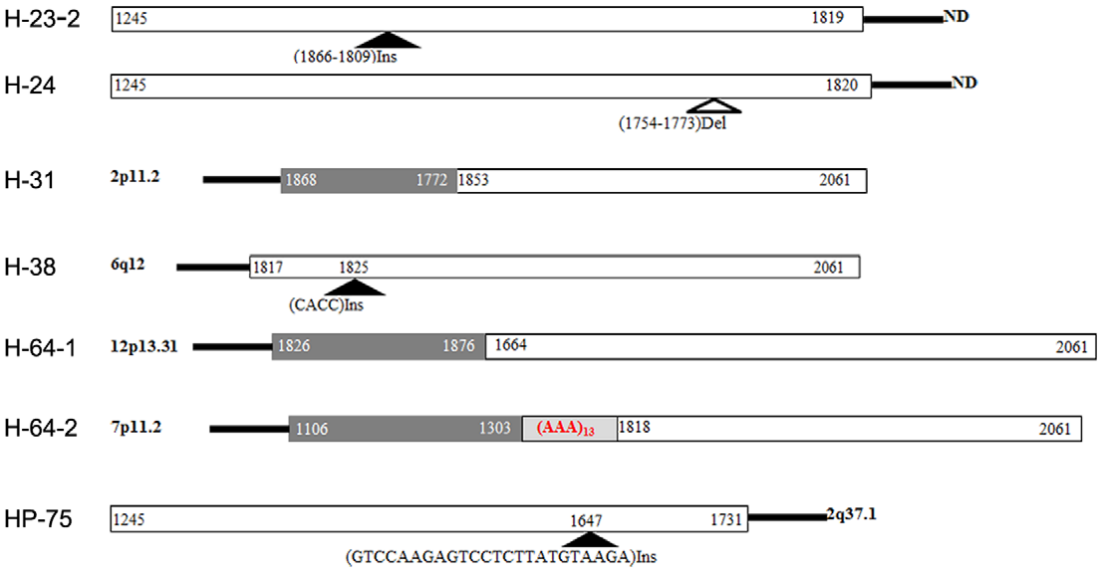
Examples of rearrangements of the HBV genome found in integrated viral genomes. Del = deletion, Ins = insertion.

Viral genome rearrangements including deletions, inversions and duplications of viral sequence in the integrants were observed (Figure 3). These rearrangements were found in 13 of 101 (12.87%) in tumor derived and 15 of 186 (8.06%) in non-tumor derived tissue respectively (File S1).

Sequencing of the host cell genome close to the viral-host junction revealed only a few micro-deletions, micro-insertions, point mutations and translocations, with no significant difference being found between tumor derived and non-tumor derived samples.

### The number of chromosome aberrations found in tumor derived samples does not correlate with HBV integration

A comprehensive aCGH assay was used to analyze host cell chromosomal abnormalities in 25 individuals from the 60 recruited patients, and selected to allow any effect of the number of HBV insertion events on chromosomal aberration to be examined. In each assay, material obtained from corresponding adjacent non-tumor tissue was used as the reference control. The number of HBV integration events detected across the 25 tumor derived samples analyzed ranged from 0 to 11 and the total number of genome aberrations (gain and loss) detected in the assay ranged from 11 to 537. No correlation was found between the number of genome aberrations identified and the number of HBV integration events in the tumor derived samples analyzed (*P* = 0.6520).

The TP53 status in tumor specements of all 60 patients was then analyzed and shown in Table S1. Twenty-one TP53 point mutations were found in 20 (33%) of the tumor derived tissues, including 20 single nucleotide missense mutations and 1 single nucleotide synonymous mutation. One sample contained 2 point mutations at TP53 19^leu^ and 24^lys^. Amongst the point mutations found 10 of them were AGG to AGT transversion at codon 249 of TP53. The majority of the point mutations were located in exon 7 (62%, 13/21), with 2 mutations in exon 2, 2 in exon 4, 2 in exon 5, and 1 in exon 8, respectively. In contrast, no mutational changes in TP53 were found in the paired adjacent non-tumor derived samples.

LOH of TP53 was successfully assayed in 51 of the 60 samples. The remaining 9 samples had no informative SNP data and therefore could not be assayed for LOH. Twenty-two of the 51(41%) patients successfully assayed showed LOH for TP53. The concurrence of TP53 point mutation and LOH was present in 20% (12/60) of the patients. Overall mutational change (point mutation and/or LOH) causing loss of TP53 function was detected in 48% (29/60) of the recruited patient cohort. No correlation was found between mutational change in TP53 and the number of HBV integration events.

Interestingly, among the 25 patients whose samples were subjected to aCGH analysis, 9 of the 11 tumor derived samples harboring TP53 mutations were found to have higher numbers of chromosomal aberrations. In contrast, 7 of the 14 tumor derived samples that had no mutations in TP53 was found lie amongst those with a higher number of chromosomal aberrations (Table 3).

**Table 3:** The distribution of HBV integration sites, mutational status of selected tumor suppressor genes and numbers of chromosomal aberrations identified in the 25 patients whose samples were assayed using aCGH assay.

Case No.	TP53 status	TP53BP2 (ASPP2)	RB1	CDKN2A	TP73	BRCA1	BRCA2	Aberration Number	HBV integration number	
									Tumor	Non-tumor
339C	W/O	gain	loss		partial-gain	gain		537	7	9
348C	LOH			loss				403	0	2
H-53	point mutation+LOH	gain		loss		gain		339	3	0
H-44	W/O		loss	loss	loss		loss	258	0	4
350C	LOH							204	0	2
H-70	point mutation		loss		loss			179	5	6
H-61	LOH	gain	loss	loss			loss	146	1	2
H-42	LOH	gain	loss					130	4	2
H-60	W/O				loss			97	11	8
H-64	W/O	gain				loss		96	6	5
H-68	W/O	loss						74	1	9
H-54	W/O	gain			loss			72	0	0
H-49	W/O				partial-gain			66	0	0
H-57	W/O							14	1	3
351C	LOH							11	2	0
197C	W/O							25	4	7
346C	W/O					loss		136	1	0
414C	W/O	gain			partial-gain			382	0	6
432C	LOH		loss		loss		loss	288	2	10
493C	W/O							126	0	0
508C	point mutation+LOH		loss		loss		loss	336	1	3
509C	W/O							176	2	7
535C	W/O	gain	loss	loss	loss		loss	235	0	2
571C	W/O							357	5	4
585C	point mutation+LOH			loss				204	2	2

**W/O = without mutation; Low aberration number = less than 100 chromosomal aberrations; High aberration number = more than 100 chromosomal aberrations.**

The mutational status of various tumor suppressor genes such as retinoblastoma 1 (RB1), TP73, cyclin-dependent kinase inhibitor 2A (CDKN2A), breast cancer 1, early onset (BRCA1), BRCA2, TP53 and TP53BP2 (tumor protein p53 binding protein, 2) in the patient samples subjected to aCGH analysis was also examined (Table 3). As expected, a positive correlation was found between the number of specific tumor suppressor gene mutations identified and the number of whole chromosomal aberrations observed (r = 0.6625, *P* = 0.0003).

## Disscussion

To date, the role of HBV integration in hepatocarcinogenesis has only been partially elucidated. The great majority of available data has been derived from the analysis of integration sites solely in tumor derived tissues [4], [10], [11], [12], [14], [18]. A systematic side by side comparative analysis of both tumor derived and non-tumor derived samples from the same patient cohort has not been reported previously.

In the present study, in agreement with a previous report, approximately 70% of tumor derived tissues were found to be carrying integrated HBV DNA [14]. A recent comprehensive HBV integration study using whole-genome deep sequencing showed that among the 3 HBV positive HCC, the number of viral integration sites was higher in tumor derived tissues compared to that in non-tumor tissues [19]. By contrast and somewhat unexpectedly, in the present study, more insertion sites were picked up in the specimens derived from the adjacent non-tumor tissues than that in the paired tumor tissues. The majority of the patients recruited to the study cohort had a cirrhotic liver back ground, in which nodule based clonal expansion is common [20]. Therefore, a possible explanation for the larger number of integration events found in our study is that DNA extracted from the adjacent non-tumor tissues may have originated from multiple cirrhotic nodules. The fact that no overlap of HBV integration sites were found between tumor derived and non-tumor derived tissue obtained from the same patient, further supported the assertion that the integration of HBV DNA occurs at random.

Previous studies have suggested that HBV DNA is preferentially inserted into known transcriptional and chromosomal regulatory regions [21]. This in turn has prompted speculation that if the gene targeted by an insertion event is tumor related, then integration would be oncogenic. However, in this study the frequencies of insertions located within known or predicted genes in tumor derived and non-tumor derived tissues were almost identical (∼42%). Indeed, the precise mapping of viral-host junctions in the present study has revealed that HBV integration occurred randomly across all human chromosomes, with no difference being observed between tumor derived and non-tumor derived samples at the chromosomal level.

The hypothesis that targeted viral genome integration might lead to ‘activation’ of cellular genes with oncogenic potential arose originally from studies on avian retroviruses [22]. Later studies on woodchuck hepatitis virus (WHV) linked WHV DNA integration with woodchuck HCC development. Integration of WHV genome sequences within the N-myc 2 gene was observed in approximately 20% of HCC tumors [23]. Furthermore based on the observed association between integration into N-myc 2 and up-regulation of the gene, it has been suggested that the up-regulation of gene expression would endow the carrier hepatocytes with a selective growth advantage and clonal expansion leading eventually to malignancy [24], [25]. In the present study, HBV insertion was found to occur in the vicinity of the oncogenic hTERT gene in 3 of the 60 HCC tumor derived tissues examined. Further analysis via realtime RT-PCR showed up-regulation of hTERT in 2 of the 3 tumor tissues, in comparison with their corresponding non-tumor tissues. These data indicated that HBV integration may enhance host gene expression, providing for the possibility that the viral insertion could be indirectly involved in hepatocyte malignant transformation by affecting host gene expression. However, to provide additional evidence in support of this possibility, deep sequencing, corresponding mRNA expression profiles and biomedical analysis will be required.

Interestingly, viral insertion was found to be significantly more frequent in the fragile sites in tumor derived tissues, than in adjacent non-tumor derived tissues. Deletion or rearrangement of genes within fragile site regions frequently occurs in a wide range of human cancers. This preference exhibited by HBV for insertion within fragile sites may induce site-specific instability and thus be a potentially oncogenic effect of HBV integration [15].

As far as the viral sequences inserted into the host genome are concerned, this study demonstrated for the first time that insertion in the HBV X gene and preC/C region was common in both tumor derived and non-tumor derived tissues. As previously reported, DR1 and the topoisomerase I motif were the preferred break-points of the inserted viral fragments in both orientations. Indeed, a recent study using DNA based- and RNA based-deep sequencing also revealed that both in tumor and non-tumor tissues, the breakpoint of inserted viral DNA clustered near DR1 region located toward the end of the HBx gene [19]. Breakage of the viral genome in this region causes C-terminal truncation of the inserted HBx and this has a high probability of resulting in the loss of the near terminal growth-suppressive effect domain of the X gene [12]. A number of *in vitro* studies have suggested that HBx carrying C-terminal truncations is more oncogenic [8]. However, we failed to show the difference in the frequency of such mutations between tumor derived and non tumor derived samples (Figure 2).

The frequencies of C1653T, T1753V and A1762T/G1764A point mutations in the X gene of the inserted HBV viral sequences were the same as that found for serum derived samples i.e.: ‘free’ non integrated viral DNA in CHB group (Table 2). This is consistent with HBV integration being an early event in the process of hepatocarcinogenesis and possibly before clonal expansion of individual tumors.

Genetic instability triggered by HBV integration has been considered in some reports to be an important contributing factor in the pathogenesis of HCC [5], [26], [27]. However, in this study the analysis of rearrangements of the integrated HBV sequences and of local alterations of the host genome surrounding integration sites failed to identify any significant discrepancies between changes found in tumor derived and non-tumor derived samples. Furthermore, in an aCGH assay, no correlation was found between HBV integration events and large-scale chromosomal alterations. Instead, a positive correlation was found between the number of aberrant tumor suppressor genes (such as TP53, RB1, TP73, BRCA1, and BRCA2) and the number of whole chromosomal aberrations observed (r = 0.6625, *P* = 0.0003). In addition, compared with the non-tumor tissues, an increased tendency of viral DNA rearrangement were found in tumor tissues (25.86% vs. 12.26%, *P* = 0.0269). However, due to limitations of PCR-based technique employed, it was not possible to evaluate the copy number changes in the vicinity of viral integration sites.

To conclude, with the exception of significantly higher frequencies of chromosome fragile sites integration and vicinal DNA rearrangement in the tumor group, all other properties of HBV insertion into the cellular genome found in this study were similar between tumor derived and adjacent non-tumor derived samples. Therefore, this control tissue validated study did not demonstrate a strong co-relationship between HBV integration and hepatocyte malignant transformation. A large scaled deep sequencing based functional study of the HBV integration in HCC patients will be needed to complete our understanding of its molecular role in HBV infection related hepatocarcinogenesis.

## Supporting Information

File S1 **The characteristics of all viral integration sites in 60 HBV positive HCC tissues and adjavent non-tumor tissues.**
(XLS)

Table S1 **The pathology data and TP53 gene status for the recruited patient cohort.**
(DOC)

Table S2 **HBV genome specific primers.**
(DOC)

Table S3 **The primers used for TP53 gene mutation screening.**
(DOC)
