# Supplementary material for: Re-evaluation of the Carcinogenic Significance of Hepatitis B Virus Integration in Hepatocarcinogenesis
Source: PLoS One. 2012 Sep 4;7(9):e40363. doi: 10.1371/journal.pone.0040363 (PMC3433482; doi:10.1371/journal.pone.0040363)
Supplement: Table S1 — The pathology data and TP53 gene status for the recruited patient cohort. (DOC) [file pone.0040363.s002.doc]

Table S1. The pathology data and the TP53 gene status for the recruited patient cohort

| Case No. | Sex | Age (Year) | Clinical stage | TP53 status |
| --- | --- | --- | --- | --- |
| 09HCC-1 | M | 54 | Ⅰ | Normal |
| 09HCC-2 | M | 64 | ⅢA | Normal |
| 09HCC-4 | F | 59 | Ⅰ | LOH |
| 09HCC-6 | M | 53 | Ⅱ | 249ser, LOH |
| 09HCC-7 | F | 51 | Ⅱ | Normal |
| 09HCC-9 | M | 53 | ⅢA | Normal |
| 09HCC-10 | M | 46 | Ⅱ | 249ser, LOH |
| 09HCC-12 | M | 37 | ⅢA | Normal |
| 09HCC-19 | M | 58 | ⅢA | Normal |
| 09HCC-21 | F | 47 | ⅢB | 249ser, LOH |
| 09HCC-23 | F | 58 | NA | 259tyr, LOH |
| 09HCC-24 | M | 57 | Ⅱ | 259tyr, LOH |
| 09HCC-26 | M | 58 | Ⅰ | 249ser, LOH |
| 09HCC-27 | M | 42 | Ⅰ | 131lys, LOH |
| 09HCC-29 | M | 57 | ⅢA | 157phe |
| 09HCC-31 | M | 46 | ⅢA | 249ser |
| 09HCC-32 | M | 60 | ⅢA | 249ser, LOH |
| 09HCC-34 | F | 37 | ⅢB | Normal |
| 09HCC-38 | M | 56 | ⅢA | Normal |
| 09HCC-39 | F | 43 | Ⅰ | 24lys, 19leu, LOH |
| 9HCC-40 | M | 54 | Ⅱ | 245asp |
| 09HCC-42 | M | 48 | ⅢA | LOH |
| 09HCC-44 | M | 48 | Ⅰ | Normal |
| 09HCC-49 | M | 65 | ⅢA | Normal |
| 09HCC-53 | M | 46 | ⅢA | 267arg, LOH |
| 09HCC-54 | M | 38 | Ⅰ | Normal |
| 09HCC-57 | M | 68 | ⅢA | Normal |
| 09HCC-58 | M | 45 | ⅢA | 249ser, LOH |
| 09HCC-60 | M | 57 | ⅢA | Normal |
| 09HCC-61 | M | 65 | ⅢA | LOH |
| 09HCC-62 | F | 42 | ⅢA | Normal |
| 09HCC-64 | M | 60 | Ⅰ | Normal |
| 09HCC-65 | M | 53 | Ⅰ | 249ser, LOH |
| 09HCC-67 | M | 49 | ⅢC | Normal |
| 09HCC-68 | M | 51 | ⅢB | Normal |
| 09HCC-69 | M | 63 | Ⅰ | Normal |
| 09HCC-70 | M | 47 | ⅢA | 249ser |
| 09HCC-73 | M | 50 | ⅢA | LOH |
| 09HCC-74 | F | 47 | ⅢA | Normal |
| 09HCC-75 | M | 55 | Ⅰ | LOH |
| 324 | M | 48 | ⅢA | Normal |
| 325 | M | 34 | Ⅱ | 68 stop codon |
| 331 | M | 44 | ⅢA | Normal |
| 336 | M | 44 | ⅢA | Normal |
| 337 | M | 47 | Ⅱ | Normal |
| 339 | M | 36 | ⅢC | Normal |
| 348 | M | 38 | ⅢC | LOH |
| 350 | M | 46 | ⅢA | LOH |
| 351 | M | 48 | Ⅱ | LOH |
| 359 | M | 41 | ⅢA | 65ser |
| 197 | F | 51 | Ⅱ | Normal |
| 346 | F | 54 | Ⅰ | Normal |
| 414 | F | 39 | Ⅱ | Normal |
| 432 | F | 70 | ⅢA | LOH |
| 493 | F | 54 | Ⅱ | Normal |
| 508 | F | 45 | Ⅱ | 249Ser, LOH |
| 509 | F | 64 | Ⅱ | Normal |
| 535 | F | 47 | Ⅱ | Normal |
| 571 | F | 49 | Ⅱ | Normal |
| 585 | F | 56 | Ⅱ | 229tyr, LOH |

M=Male, F=Female, LOH = loss of heterozygosity.
