# Supplementary material for: Re-evaluation of the Carcinogenic Significance of Hepatitis B Virus Integration in Hepatocarcinogenesis
Source: PLoS One. 2012 Sep 4;7(9):e40363. doi: 10.1371/journal.pone.0040363 (PMC3433482; doi:10.1371/journal.pone.0040363)
Supplement: Table S2 — HBV genome specific primers. (DOC) [file pone.0040363.s003.doc]

Table S2. HBV genome specific primers

| Primers | Location in  HBV genome | Sequence (5’-3’) |
| --- | --- | --- |
| HBV1  HBV2 | 2061-2039 | GCUGUAUGGUGAGGUGAACAAUG |
|  | 2054-2019 | GGTGAGGTGAACAATGTTCCGGAGACTCTAAGGCCT |
| HBV3 | 1262-1285 | CGATCCATACTGCGGAACTGCTAG |
| HBV4 | 2342-2364 | GACCTGCCTCGTCGTCTAAGAAC |
