# Supplementary material for: Re-evaluation of the Carcinogenic Significance of Hepatitis B Virus Integration in Hepatocarcinogenesis
Source: PLoS One. 2012 Sep 4;7(9):e40363. doi: 10.1371/journal.pone.0040363 (PMC3433482; doi:10.1371/journal.pone.0040363)
Supplement: Table S3 — The primers used for TP53 gene mutation screening. (DOC) [file pone.0040363.s004.doc]

Table S3. The primers used for TP53 gene mutation screening

| PCR* products | Primer sets** | Sequence (5’-3’) |
| --- | --- | --- |
| TP53 exon 2-4 | 10815F | GCTGTCTCAGACACTGGCATGGT |
|  | 11604R | TACGGCCAGGCATTGAAGTCTC |
| TP53 exon 5-6 | 11940F | CCTGGGTAACATGATGAAACCTCGTC |
|  | 12793R | AGGTCAAATAAGCAGCAGGAGAAAGC |
| TP53 exon 7-10 | 13147F | GGCGACAGAGCGAGATTCCATCTCA |
|  | 14403R | AGCAGGCTAGGCTAAGCTATGATGTT |
| TP53 exon 11-12 | 16736F | TTGTACCGTCATAAAGTCAAACAAT |
|  | 18076R | ACAAAGCAAATGGAAGTCCTGGGTG |

*The PCR conditions were as follows: after denaturation at 95℃ for 2 min, PCR consisted of 40 cycles of 20s at 95℃, 20s at 59℃ to 67℃, and 30s to 50s at 72℃, subsequently followed by a final extension step of 5 min at 72℃.

**The location of primers were based on NM_001126112.2.
